# Enhancing lucerne (*Medicago sativa*) yield and nutritional quality: a meta-analysis of fertilization types and environmental factors in China

**DOI:** 10.3389/fpls.2024.1405180

**Published:** 2024-07-09

**Authors:** Jiachang Zhang, Yanting Mao, Gang Wang, Dong Luo, Quan Cao, Kadambot H. M. Siddique, Morad Mirzaei, Matthew Saunders, Fateme Aghamir, Emanuele Radicetti, Yangzhou Xiang, Qingping Zhang, Yuan Li, Yuying Shen

**Affiliations:** ^1^ The State Key Laboratory of Herbage Improvement and Grassland Agro-Ecosystems, National Field Scientific Observation and Research Station of Grassland Agro-Ecosystems in Gansu Qingyang, College of Pastoral Agriculture Science and Technology, Lanzhou University, Lanzhou, China; ^2^ Institute of Agricultural Environment and Resources, Yunnan Academy of Agricultural Sciences (YAAS), Kunming, China; ^3^ Agricultural Research Institute of Jiuquan, Jiuquan, Gansu, China; ^4^ Academy of Animal and Veterinary Science, Qinghai University, Xining, China; ^5^ The UWA Institute of Agriculture, The University of Western Australia, Perth, WA, Australia; ^6^ School of Natural Sciences, Botany Discipline, Trinity College Dublin, Dublin, Ireland; ^7^ Environmental Sciences Research Institute, Shahid Beheshti University, Tehran, Iran; ^8^ Department of Chemical, Pharmaceutical and Agricultural Sciences (DOCPAS), University of Ferrara, Ferrara, Italy; ^9^ School of Geography and Resources, Guizhou Provincial Key Laboratory of Geographic State Monitoring of Watershed, Institute of Guizhou Mountain, Guizhou Education University, Guiyang, China; ^10^ College of Agriculture and Forestry Science, Linyi University, Linyi, China; ^11^ Grasslands and Sustainable Farming, Production Systems Unit, Natural Resources Institute Finland, Kuopio, Finland

**Keywords:** alfalfa, fertilization practices, yield improvement, nutritional quality, environment factors, agricultural practices, meta-analysis

## Abstract

**Introduction:**

Lucerne (*Medicago sativa*), is a cornerstone of China’s livestock industry, however, due to the backward agronomic strategies and technology, lucerne in China faces cultivation challenges that result in lower productivity and quality than global standards. Therefore, we undertook a meta-analysis to evaluate the impact of five distinct fertilization types on lucerne yield and nutritional quality in various locations in China. The fertilizer practices included manure application, combined mineral fertilizer and manure application (FM), biological fertilizer application, unbalanced application of two or more mineral fertilizer types, and balanced mineral fertilizer application. Furthermore, we investigate influential factors of yield and quality of lucerne under fertilization, including climatic variables (mean annual precipitation, mean annual temperature), initial soil properties (soil organic carbon; total nitrogen, pH), and agronomic factors (seeding rate, harvest frequency, and lucerne stand age).

**Methods:**

Our study analyzed 53 published papers to discern the most beneficial fertilizer for enhancing lucerne yield and nutritional quality.

**Results and discussion:**

The results showed that the fertilizer practices, on average, significantly improved yield by 31.72% and crude protein content by 11.29%, with FM emerging as the most effective, this is because mineral fertilizers provide available nutrients for lucerne, manure provides essential organic matter for microorganisms and improve soil properties. In addition, the fertilizer practices significantly reduced neutral and acid detergent fiber contents by 6.28% and 8.50%, respectively, while increasing ash content and relative feeding value. Furthermore, climatic variables, soil properties, and planting system factors such as sowing date and harvest frequency significantly affected yield and nutritional quality. The practical implications of our results emphasize the need for balanced and strategic fertilizer application to optimize lucerne production and highlight the potential to adjust cultivation practices according to environmental conditions. Balanced and strategic fertilizer application can simultaneously improve soil properties, enhance soil carbon sequestration, and reduce the emission of greenhouse gases from the soil, which is a vital measure for realizing sustainable agricultural development.

## 1 Introduction

Lucerne (*Medicago sativa* L.), a perennial forage crop known for its high protein content, excellent digestibility, and resilience to environmental stresses, holds the title of ‘Queen of Forage’. This high regard stems from its remarkable yield, protein content, and other nutritional benefits (Palmonari et al., 2014; Teixeira et al., 2023). In China, where lucerne has established itself as the leading leguminous forage, its importance cannot be overstated (Stritzler et al., 2018; Acharya et al., 2020; Wang and Zhong, 2021). However, its cultivation is fraught with challenges arising from a variety of environmental factors and inconsistent agronomic practices, including fluctuating fertilization strategies and field protocols. These inconsistencies often lead to suboptimal yields and a decrease in nutritional quality, issues that are particularly prominent in China (Lin et al., 2017; Feng et al., 2022).

Despite its widespread cultivation, the average yield and quality of lucerne per hectare remain low in China. For instance, while China produced 32.17 million tons of lucerne hay in 2015, only 1.8 million tons met the standards for high-quality lucerne, which typically has high crude protein (CP) content and digestibility (Ta et al., 2020). This pales in comparison to the USA, where the production of high-quality lucerne hay reached 52.60 million tons (Wang and Zhong, 2021). Consequently, China relies heavily on imported lucerne hay, and nearly a quarter of China’s lucerne demand is still met through imports (Wang, 2022), with China importing a staggering 509.1 million tons of lucerne hay from the United States between 2017 and 2020 (Wang and Zhong, 2021).

As a legume forage, lucerne has a strong nitrogen (N)-fixation root nodule system. Yet, long-term lucerne cultivation also depletes other essential soil nutrients (Fang et al., 2021). Specifically, deficiencies in either phosphorus (P) or potassium (K) can significantly hinder lucerne’s yield, forage quality, and environmental adaptability (Malhi, 2011; Yahaya et al., 2023). Effective agronomic strategies, such as appropriate nutrient application, are needed to enhance lucerne’s yield and nutritional quality. Nevertheless, 43% of farmers still did not apply fertilizers for alfalfa production in the main alfalfa production areas in China according to a survey conducted in 2013 (Kai-Yun et al., 2016). Meanwhile, excess or irrational fertilization would lead to bad consequences (Good and Beatty, 2011). Empirical evidence further supports the benefits of fertilization in lucerne cultivation, including increased yield and CP content (Hakl et al., 2016), reduced neutral (NDF) and acid detergent fiber (ADF) contents (Macolino et al., 2013), and enhanced relative feeding value (RFV) (Jungers et al., 2019; Zhao et al., 2022).

Despite considerable research on lucerne cultivation, including studies on water stress (Dhakal et al., 2020; Wang et al., 2021) and P and K fertilization (Berg et al., 2007; Lissbrant et al., 2009), the study to identify the optimal fertilizer combination for lucerne in China continues. Previous efforts have significantly improved our understanding of how various fertilization types, especially combined organic and inorganic fertilizers, impact soil organic carbon and total N contents, thereby enhancing soil health, microbial activity, and nutrient availability (Kas et al., 2016; Wang et al., 2021; Yahaya et al., 2023). These improvements are crucial for lucerne’s growth and development. Because of the higher abundance of beneficial microbes is positively related the higher soil quality, including better plant growth, lower disease incidence, and higher nutrient contents, soil enzyme activities and soil pH (Wang et al., 2017). Earlier meta-analyses have primarily focused on the yield effects of fertilization alone, considering factors like growing ages (Cai et al., 2020, 2021); or yield and quality responses to fertilization in relation to precipitation (Ye et al., 2023) or initial soil organic matter contents (Wan et al., 2022). Previous research on lucerne fertilization has primarily focused on the direct effects of various fertilizers on yield and quality. However, these studies often overlook the intricate interactions between environmental and management factors that also play crucial roles. Further study is warranted to investigate the interplay between climate variables, soil properties, agronomic practices, and different fertilization types, collectively influencing lucerne yield and quality. This comprehensive approach is critical for developing more effective fertilization strategies that are tailored to specific environmental conditions and management practices, thereby optimizing both the productivity and nutritional quality of lucerne.

The objective of our study was to conduct a meta-analysis of the available literature, focusing on the impact of different types of fertilizers on lucerne yield and quality characteristics in China. We aimed to identify the most effective fertilization strategies, considering the yield and nutritional value of lucerne. Moreover, this study incorporated environmental and agronomic variables, thereby providing a more holistic understanding of how these factors interact with various fertilization types. Relevant observation will determine the optimal fertilization strategy to improve the yield and quality of lucerne, reduce the dependence on imports of lucerne in China, and contribute to the sustainability of agriculture, food security and economic development in China.

## 2 Materials and methods

### 2.1 Literature and data collection

We conducted a comprehensive literature search using the China Knowledge Resource Integrated Database (http://www.cnki.net/) to identify relevant studies on the effects of fertilization on lucerne yield and nutritional quality, published between 1990 and June 2022. The search terms included combinations of the following keywords: ‘fertilization,’ ‘fertilizer,’ ‘organic,’ ‘manure,’ ‘mineral,’ ‘synthetic,’ ‘alfalfa,’ ‘*Medicago sativa*,’ ‘lucerne,’ ‘quality,’ ‘nutrition,’ ‘nutritive value,’ ‘crude protein,’ ‘feed value,’ and ‘detergent fiber’.

**Figure 1:**
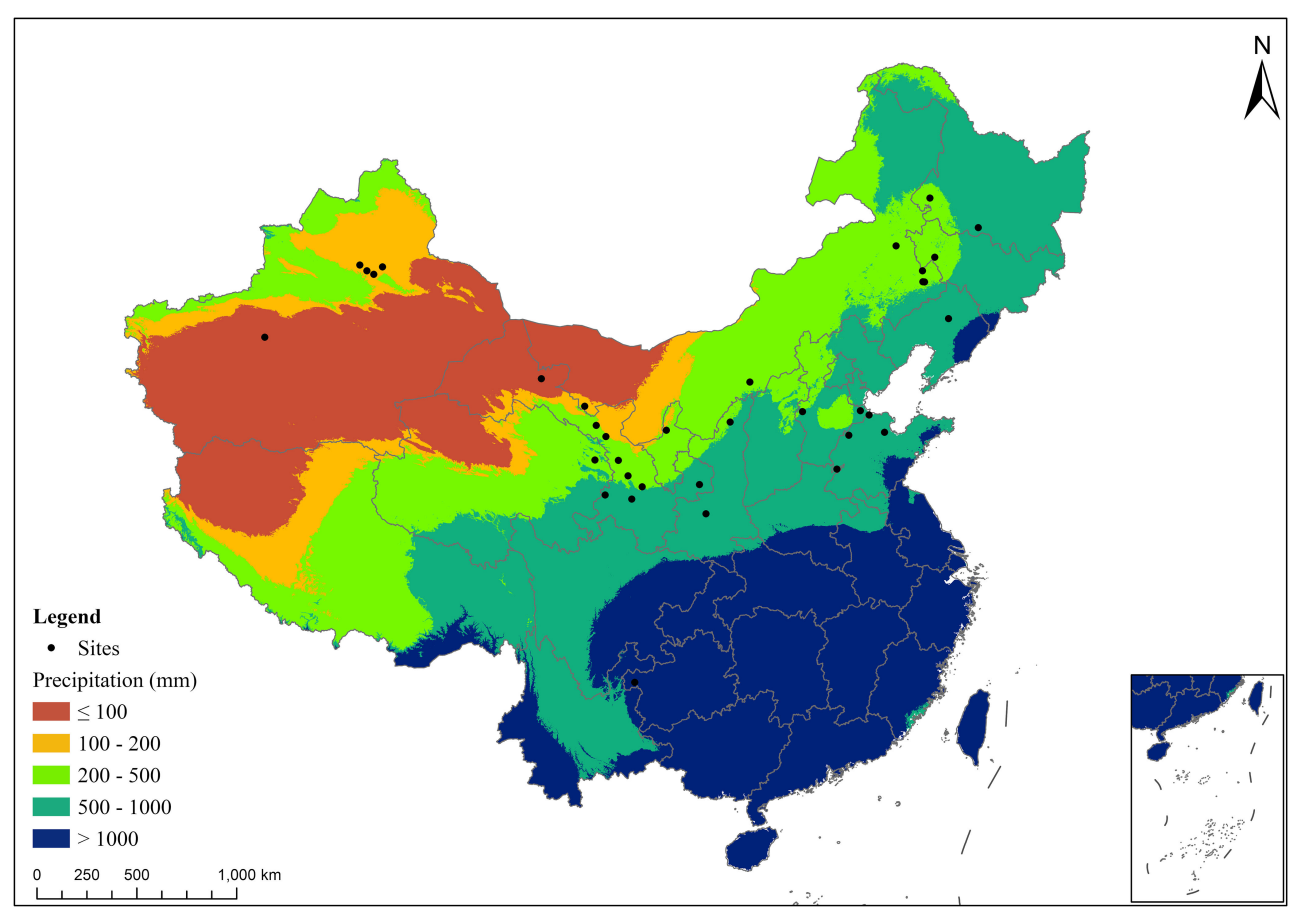
The mean annual precipitation of the studies used in the meta-analysis (the color version of the figure is in the web version of this article).

Following the initial search, we undertook a thorough screening process to refine the selection based on the following criteria: (1) the experimental study was conducted in China, with the test site and duration stated in the study; (2) the study included at least one control group (with no fertilization) and one group receiving fertilization treatment. Five fertilization types were considered (**Table 1**): manure application (M), combined mineral fertilizer and manure application (FM), biological fertilizer application (BioF), unbalanced application of two or more mineral fertilizer types (UCF), and balanced mineral fertilizer application (CF). These categories were selected to cover a broad spectrum of common fertilization practices, thereby enabling a comprehensive assessment of their effects on lucerne’s yield and nutritional quality. For studies including other fertilization treatments, data were extracted solely from these five fertilization types; (3) the study directly reported lucerne yield and nutritional quality measures, such as crude protein (CP), ether extract (EE), neutral (NDF) or acid detergent fiber (ADF), relative feeding value (RFV), and ash content; (4) All other treatment variables (e.g., irrigation, tillage) were consistent between the control and treatment groups. Studies involving root fertilization and foliar topdressing were excluded. After the selection process, relevant data were extracted from the chosen studies. Data were extracted from charts using Get-Data Graph Digitizer (http://getdata-graph-digitizer.com/) or directly from tables or text in the articles. We identified 53 papers for inclusion in our meta-analysis (**Figure 1**). Information about the experimental site, climatic variables, initial soil properties, and yield and nutritional quality variables involved in this meta-analysis were summarized in **Tables 2** and **3**.

**Table 1:** Description of fertilization types in the meta-analysis.

Fertilization description	Abbreviation
Unbalanced mineral fertilization of N, P, or K	UCF
Balanced mineral fertilization of N, P, and K	CF
Biological fertilizer	BioF
Manure and mineral fertilizers	FM
Manure only	M

Fertilizers include nitrogen (N), phosphorus (P), and potassium (K); UCF is the unbalanced application of one or two types of mineral fertilizer (N only, P only, K only, N and P, N and K, and P and K); CF is the balanced mineral fertilization of N, P, and K.

**Table 2:** Summary of site information, climatic variables, and initial soil properties for the meta-analysis.

	MAP (mm)	MAT (°C)	SOC (g kg^–1^)	TN (g kg^–1^)	AN (mg kg^–1^)	AP (mg kg^–1^)	AK (mg kg^–1^)	pH	Seeding rate (kg ha^–1^)	Age (years)
Min	59.80	0.00	1.40	0.26	6.15	1.95	11.40	6.44	11.00	1.00
Max	859.00	14.30	14.02	1.80	135.00	136.00	478.00	8.80	30.00	8.00
Median	350.00	6.70	5.86	0.76	50.00	13.80	100.00	8.10	22.50	2.00
Mean	379.00	7.73	6.96	0.82	53.40	25.10	125.00	8.01	21.00	2.06
SD	176.00	2.78	3.67	0.38	28.20	27.10	75.00	0.44	5.29	1.47
CV	0.46	0.36	0.53	0.47	0.53	1.08	0.60	0.06	0.25	0.71

MAT, mean annual temperature; MAP, mean annual precipitation; SOC, soil organic carbon; TN, soil total nitrogen; AN, soil available nitrogen; AP, soil available phosphorus; AK, soil available potassium; Age, age of lucerne stand; SD, standard deviation; CV, coefficient of variation.

**Table 3:** Summary of variables in the meta-analysis.

	Yield	CPY	CP	ADF	NDF	RFV	Ash	EE
Min	–0.08	–0.05	–0.99	–0.56	–0.43	–0.10	–2.06	–3.23
Max	1.49	1.76	0.79	0.26	0.47	0.65	0.94	1.27
Median	0.27	0.37	0.08	–0.07	–0.06	0.12	0.03	0.07
Mean	0.32	0.46	0.10	–0.09	–0.06	0.13	0.04	0.04
SD	0.23	0.34	0.15	0.11	0.11	0.12	0.19	0.33
CV	0.74	0.74	1.40	–1.29	–1.69	0.89	4.56	7.95

Weighted response ratio (*RR*_++_) of lucerne yield, CP, CPY, EE, ADF, NDF, RFV, and ash content with fertilizer addition across China. CPY, crude protein yield; CP, crude protein; ADF, acid detergent fiber; NDF, neutral detergent fiber; RFV, relative feed value; ash content and EE, ether extract; SD, standard deviation; CV, coefficient of variation.

Refer to **Figure 3** caption for descriptions of the abbreviations.

### 2.2 Data analysis

The natural logarithmic response ratio (ln*RR*) was used to quantify the magnitude of the treatment effect of fertilizer addition on lucerne yield and nutritional quality. The ln*RR* was calculated as: ln*RR* = ln (*X*_t_/*X*_c_), where *X*_t_ and *X*_c_ are the means of lucerne yield or nutritional quality parameters for the fertilization treatments and the control (no fertilization), respectively.

The variance (*v*) of ln*RR* was calculated as Equation 1:


(1) $$v=\;S_{t}{\;}^{2}/n_{t}\times \;X_{t}{\;}^{2}+\;S_{c}{\;}^{2}/n_{c}\times \;X_{c}{\;}^{2}$$

where *S*_t_ and *S*_c_ are the standard deviations (sd) and *n*_t_ and *n*_c_ are the number of replicates for the treatment and the control groups, respectively.

To derive the overall response effects of the treatment groups relative to the control group, the weighted mean response ratio (*RR*_++_) and standard deviation of *RR*_++_ (s(*RR*_++_)) from each *RR* were calculated as Equations 2–4:


(2) $$RR_{++}=\frac{\sum_{i=1}^{m}\sum_{j=1}^{k}w_{ij}RR_{ij}}{\sum_{i=1}^{m}\sum_{j=1}^{k}w_{ij}}$$

(3) $$\text{s}\left({\text{RR}}_{\text{++}}\right)=\sqrt{\frac{1}{\sum_{\text{i}=1}^{\text{m}}\sum_{\text{j}=1}^{\text{k}}{\text{w}}_{\text{ij}}}}$$

(4) w= 1/v

where m is the number of compared groups, *k* is the number of comparisons in the corresponding groups, *w_ij_* is the weighting coefficient, and *i* and *j* are the *i*-th and *j*-th treatment groups, respectively.

The 95% confidence interval (95% CI) was calculated as 95%CI = *RR*_++_ ± 1.96 × s(*RR*_++_). If the 95% CI did not overlap zero, the fertilization treatment effects significantly differed from the control group. For ease of interpretation, the percentage change between the treatment and control was calculated as (exp(*RR*_++_) - 1) × 100%.

The influence of site-specific factors, such as climatic variables, initial soil properties, and agronomic factors on changes in lucerne yield and nutritional quality with fertilizer addition were examined. Climatic Variables: Mean annual precipitation (MAP, mm): sourced from local meteorological data corresponding to the study sites reported in the reviewed articles; Mean annual temperature (MAT, °C): obtained from meteorological data associated with the study locations. Initial Soil Properties: Soil organic carbon (SOC, g kg^-1^): data derived from soil tests reported in the studies or local soil surveys; Total nitrogen (TN, g kg^-1^): information collected from soil analyses provided in the research articles; pH: representing the acidity or alkalinity of the soil, with data taken from soil test results included in the reviewed literature. Agronomic Factors: Seeding rate: Measured in kilograms per hectare (kg ha^-1^), values extracted from the experimental setup sections of the studies; Harvest frequency: Reported as the number of cuts per growing season, with specifics depending on the agricultural practices documented in each study; Lucerne stand age: Measured in years, indicating the age of the lucerne crop at the time of data collection as reported in the studies. A heat map of the correlations between the *RR* of lucerne and the environmental/agronomic factors was generated, and Pearson’s correlation analysis was performed using the corrr package (Jackson et al., 2016) in R 4.1.1. To analyze the impact of various factors on the *RR* of lucerne yield, CP, and RFV across China, we employed Classification and Regression Trees (CART) and the Random Forest Model. CART analysis helped identify and illustrate the hierarchical influence of factors such as fertilizer type, climate, and soil properties on lucerne’s performance. This method involves recursively partitioning the data set into subsets, which are then analyzed within their specific context (Izenman, 2008). Meanwhile, the Random Forest Model provided a robust estimation of the importance of these factors by constructing a multitude of decision trees and using the mean decrease in accuracy (IncMSE %) to rank their predictive importance (Liaw and Wiener, 2002).

## 3 Results

### 3.1 Effects of fertilizer application on lucerne yield and quality

**Figure 2:**
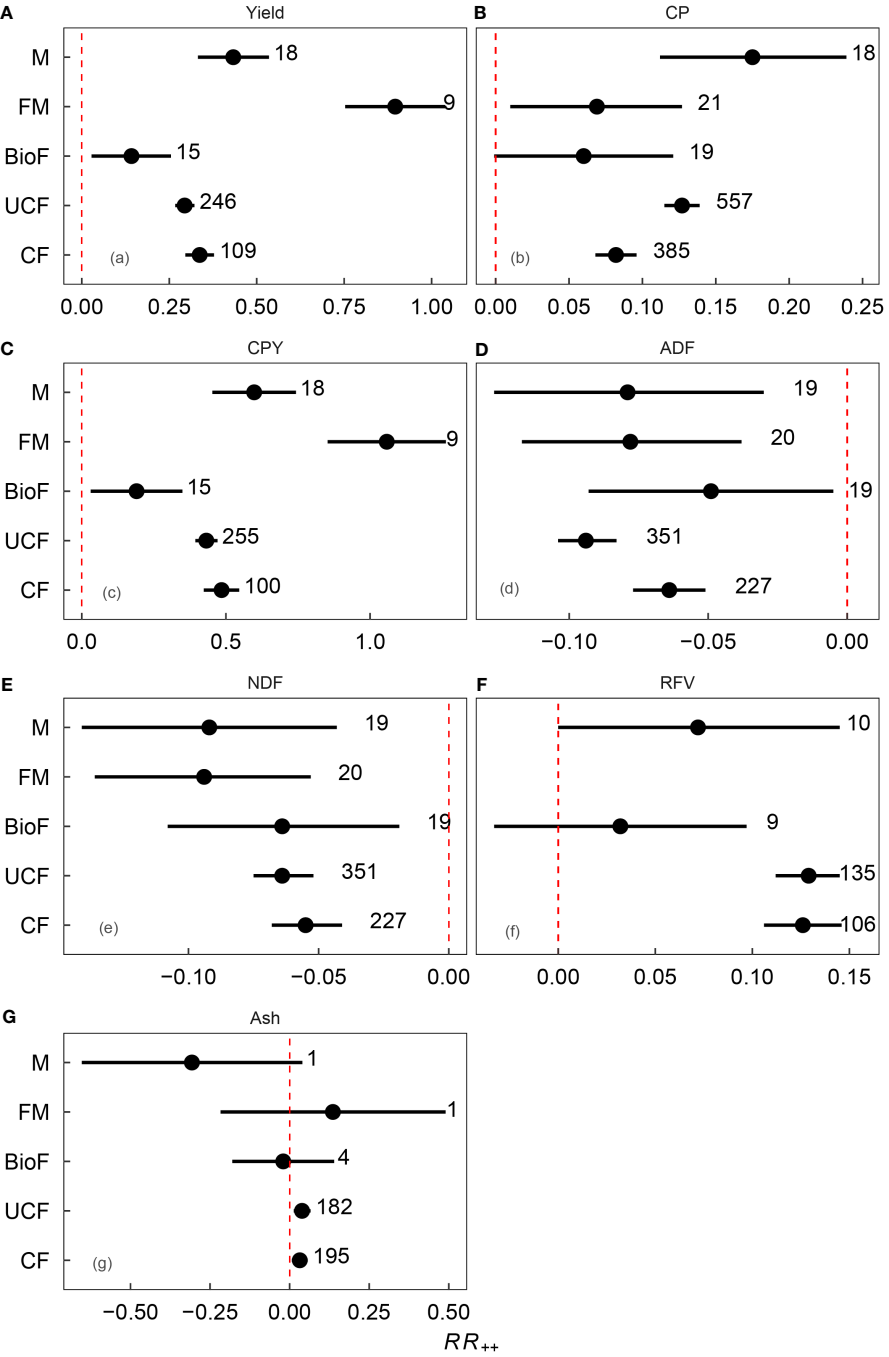
Weighted response ratio (*RR*_++_) of lucerne **(A)** yield, **(B)** crude protein (CP), **(C)** crude protein yield (CPY), **(D)** acid detergent fiber (ADF), **(E)** neutral detergent fiber (NDF), **(F)** relative feed value (RFV), and **(G)** ash content under different fertilizer types. Circles and error bars represent the means and 95% confidence intervals. Red dashed line indicates no change in nutritional quality in response to fertilizer addition compared to the control. *RR*_++_ values were considered significant if the 95% confidence interval did not overlap 0. The number of studies and observations are listed in parentheses. Refer to **Table 1** for descriptions of the abbreviations.

**Figure 3:**
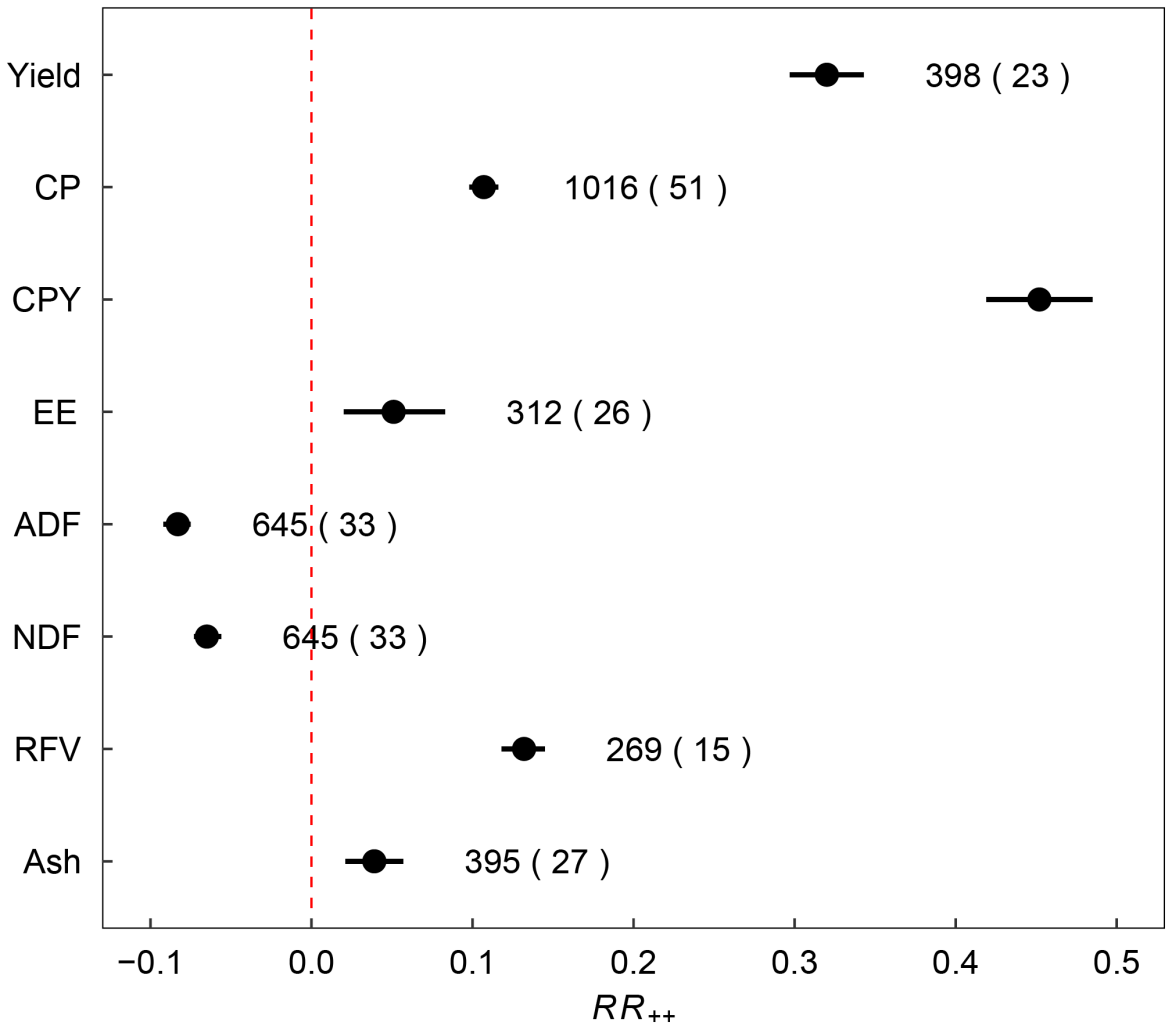
Weighted response ratio (*RR*_++_) of lucerne yield, crude protein (CP), crude protein yield (CPY), ether extract (EE), acid detergent fiber (ADF), neutral detergent fiber (NDF), relative feed value (RFV), and ash content with fertilizer addition across China. Circles and error bars represent the means and 95% confidence intervals. Red dashed line indicates no change in nutritional quality in response to fertilizer addition compared to the control. *RR*_++_ values were considered significant if the 95% confidence interval did not overlap 0. The number of studies and observations are listed in parentheses.

Fertilizer application had a multifaceted impact on lucerne yield and its quality parameters. Specifically, the application of fertilizers significantly enhanced lucerne yield by 31.72%. However, the magnitude of the impact varied: UCF, CF, BioF, FM, M increased lucerne yield by 29.4%, 33.7%, 14.2%, 89.6% and 43.3%, respectively (**Figure 2**). Furthermore, the application of fertilizers also changed the quality trait of lucerne, which increased CP, CPY, EE, RFV, and ash content by 11.29%, 45.61%, 4.18%, 14.11%, and 4.19%, respectively. In contrast, it significantly reduced ADF and NDF by 6.28% and 8.50%, respectively, compared to the control (**Figure 3**). Differential impacts were observed when various types of fertilizers were applied. For instance, the FM treatment exhibited the most potent effect, boosting CPY by 105.80% (*p*<0.05). In contrast, BioF treatment generated the least pronounced effect, enhancing CPY by 19.00% (*p*<0.05, **Figure 2C**). All fertilizer types except BioF led to an increase in CP content, with M and UCF surpassed the CF treatment (*p*<0.05; **Figure 2B**). The CF treatment was most effective in reducing ADF compared to UCF treatment (*p*<0.05, **Figures 2D, E**). For RFV and ash content, significant positive impacts were identified under UCF (12.90% and 3.98%) and CF (12.60% and 3.25%) treatments (**Figures 2F, G**).

**Figure 4:**
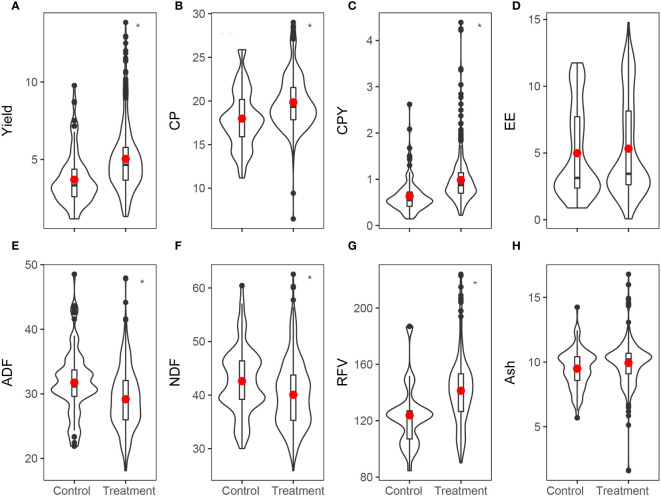
Overview of lucerne **(A)** yield (t ha^–1^, n=398), **(B)** crude protein (CP, %, n=1016), **(C)** crude protein yield (CPY, t ha^–1^, n=398), **(D)** ether extract (EE, %, n=307), **(E)** acid detergent fiber (ADF, %, n=636), **(F)** neutral detergent fiber (NDF, %, n=636), **(G)** relative feed value (RFV, %, n=636), and **(H)** ash content (%, n=390) between control and fertilizer treatment. Horizontal line and red dot indicate the median and average values, box limits represent the 25^th^ and 75^th^ percentiles (lower and upper limits, respectively), and vertical bars represent the 5^th^ and 95^th^ percentiles. Note the different scales between graphs. Statistical significance between groups are indicated by * at 0.05 level.

Overall, the application of fertilizers led to a significant increase in the *RR* of yield and quality indicators such as CP, CPY, and RFV (**Figures 4A–C, G**). Concurrently, it resulted in a significant decrease in *RR* of ADF and NDF contents (**Figures 4E, F**). The *RR* of EE and ash content contents remained relatively unchanged (*p*>0.05, **Figures 4D, H**).

### 3.2 Interaction effects among fertilizer and different environmental factors on lucerne yield and quality

**Figure 5:**
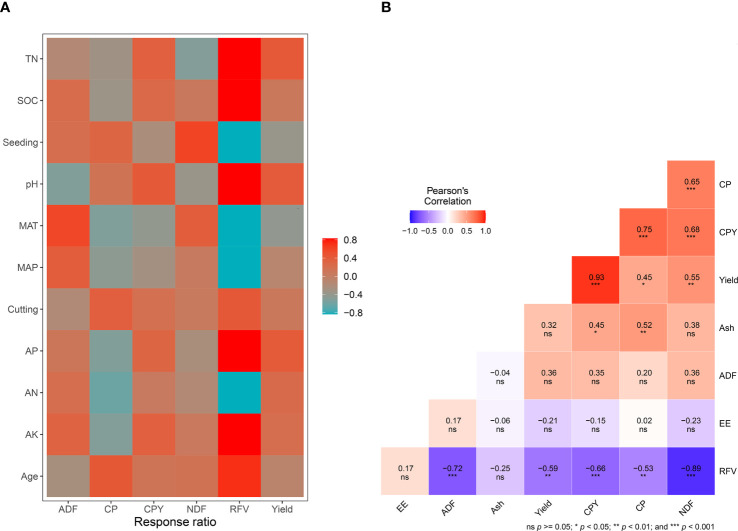
**(A)** Pearson’s correlations between the response ratio (control vs. treatment) of yield, or nutritional quality and climate [mean annual precipitation (MAP), mean annual temperature (MAT)], initial soil properties [initial soil organic carbon (SOC), initial soil pH (pH), initial soil total nitrogen (TN), initial soil available nitrogen (AN), initial soil available potassium (AK), and initial soil available phosphorus (AP)], and agronomic factors [lucerne stand age, seeding rate, harvest frequency (cutting)]; **(B)** Correlation between logarithmic response ratio (ln*RR*) of lucerne yield or nutritional quality with fertilizer addition across China. Nutritional quality indexes include crude protein (CP), crude protein yield (CPY), acid detergent fiber (ADF), neutral detergent fiber (NDF), relative feed value (RFV), and ash content. Correlation coefficients are indicated by numbers with color gradients (the color version of the figure is in the web version of this article).

The environmental and agronomic factors showed significant correlations with various properties of lucerne when fertilizer was applied (**Figure 5A**). MAT had a significant influence on lucerne yield, CPY, CP, ADF, and RFV, with absolute correlation coefficients (r) exceeding 0.4. Soil TN, AP, and soil pH were positively correlated with lucerne yield, with *r* values of 0.42, 0.41, and 0.40, respectively (*p*<0.05). These soil variables were further analyzed to discern which type of fertilizer might be more effective in locations with these specific soil properties.

The role of MAP, lucerne stand age, AN, AP, and AK in affecting the CP content of lucerne were also examined (**Figure 5A**), with significant correlations observed (*p*<0.01). The NDF content in lucerne was influenced by TN and seeding rate (*r* values of -0.50 and 0.60, respectively; *p*<0.01).

An extensive cross-correlation analysis among various quality parameters of lucerne under different fertilizer treatments was also performed (**Figure 5B**). CPY significantly correlated with ash content (*r*=0.45). Lucerne yield significantly correlated with RFV and NDF (*r*= –0.59 and 0.55), and CP correlated with RFV and ash (*r*= –0.53 and 0.52; *p*<0.01). RFV had highly significant negative correlations with ADF, CPY, and NDF (*r*= –0.72, –0.66, and –0.89; *p*<0.001), and CPY had highly significant positive correlations with NDF (*r*= 0.68). Highly significant correlations also occurred between CP and NDF (*r*=0.65).

**Figure 6:**
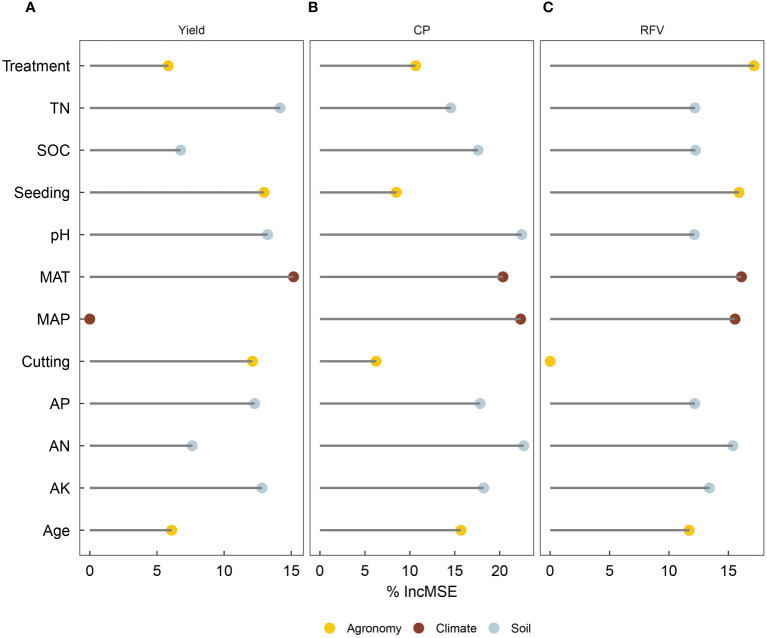
Relative importance of independent variables in predicting the response ratio (control vs. treatment) of lucerne **(A)** yield, **(B)** crude protein (CP), and **(C)** relative feed value (RFV) across China. Note different x-axis scales among graphs. Random forest analysis was used to determine the percent increase in mean squared error (%IncMSE) when each variable was randomized, indicating higher importance with greater %IncMSE. Variables include fertilizer type, climate [mean annual precipitation (MAP)], initial soil properties [initial soil pH (pH), initial soil total nitrogen (TN), initial soil available nitrogen (AN), and initial soil available phosphorus (AP)], and agronomic factors [lucerne stand age, seeding rate, harvest frequency (cutting)].

**Figure 7:**
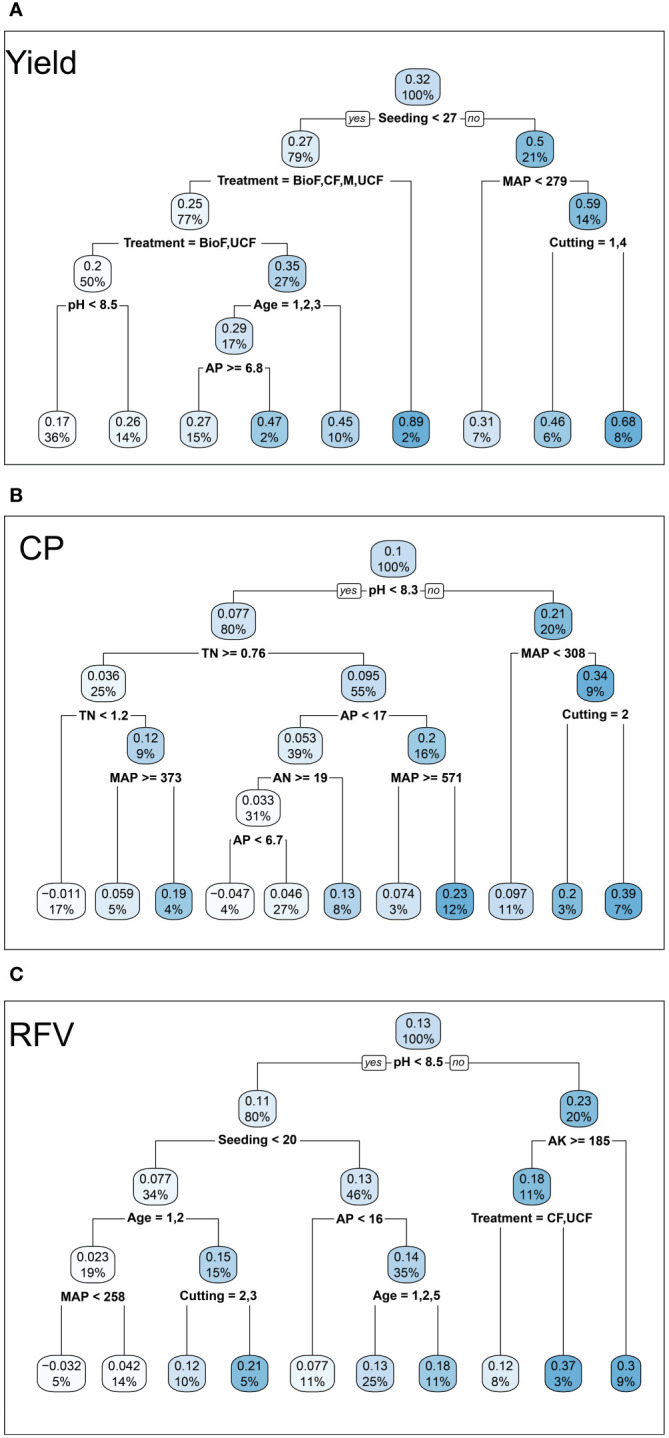
Classification and regression trees illustrated the influence of fertilizer type, climate [mean annual precipitation (MAP)], initial soil properties [initial soil pH (pH), initial soil total nitrogen (TN), initial soil available nitrogen (AN), and initial soil available phosphorus (AP)], and agronomic factors [lucerne stand age, seeding rate, harvest frequency (cutting)] on the response ratio (control vs. treatment) of lucerne **(A)** yield, **(B)** crude protein (CP), and **(C)** relative feed value (RFV) across China. The numbers and the shading in the boxes represent the mean value at each decision point; the percentages indicate the percentage of all values considered at that decision point.

Climate, initial soil TN, and pH were important for changes in the *RR* of yield, followed by seeding rate (**Figure 6A**). Climate, initial soil pH, and AN were important for changes in the *RR* of yield (**Figure 6B**). Fertilizer type, climate, and seeding rate were important for changes in the *RR* of RFV, followed by initial soil AN (**Figure 6C**). This was consistent with the outputs of regression trees, indicating the initial split node (**Figure 7**).

## 4 Discussion

### 4.1 Efficacy of different fertilizer types in enhancing lucerne yield and quality

Strategic fertilizer management can significantly improve lucerne yield and quality. This meta-analysis confirms previous findings that long-term treatment without fertilizer leads to a decline in lucerne dry matter yield, while manure and mineral fertilizer applications can increase yield (Hakl et al., 2016). Our results provide further evidence by demonstrating the positive impact of five distinct fertilizer types on lucerne yield and quality (**Figures 3**, **4**). Lucerne yield and protein production significantly increased with fertilizer application, consistent with previous studies reporting positive effects of N and P fertilizer application on lucerne yield and protein content, while K fertilizer improved yield but reduced crude protein yield (Lissbrant et al., 2009). In general, the reduction in crude protein yield with K fertilizer application could be attributed to increased branch numbers at the expense of the leaf-to-shoot ratio (Hakl et al., 2021; Yahaya et al., 2023). Furthermore, the greater height and yield of lucerne may lead to fierce competition for light, which can explain the lower CP content of lucerne in response to K fertilizer application (Sheaffler et al., 1986; Lemaire et al., 1991; Macolino et al., 2013).

Among the different fertilization types, the combined mineral and manure fertilizer application (FM) performed best in the meta-analysis. This synergistic effect is likely due to the complementary nutrient profiles provided by both organic and inorganic sources, creating optimal conditions for robust plant growth (Ananda et al., 2021). Meanwhile, this result is in line with the previous observation, combined mineral and manure fertilizer application could improve the yield and quality of plants better than other types of fertilization, this could be attributed to the increases in soil multifunctionality and microbial richness, and treatment with manure fertilizer can mitigate the decrease in genes for C-cycling function caused by the long-term application of mineral fertilizer (Ying et al., 2023). This observation might also suggest the importance of P and/or K application in enhancing the yield and quality of lucerne. Specifically, the increased availability of P was found to correlate with improved root development and more efficient utilization of N and other nutrients, which is reflected in the increased CPY (Kong et al., 2020; Hakl et al., 2021; Wan et al., 2022). Furthermore, the application of K fertilizer may increase water-use efficiency by accelerating the growth of plant roots, which aligns with K’s role in enhancing plant stress tolerance and overall vigor, contributing to the enhanced yield and quality of lucerne (Kong et al., 2020; Hakl et al., 2021; Yahaya et al., 2023). In addition to P and K, the role of microelements in manure could also emerge as a significant role in enhancing the yield and quality of lucerne. Microelements were found to be instrumental in various physiological processes including enzyme activation, photosynthesis, and N metabolism (Kong et al., 2020; Hakl et al., 2021).

### 4.2 Various responses of nutritional quality to different fertilization types

Our analysis of five fertilization types (M, FM, BioF, UCF, CF) revealed their significant role in enhancing lucerne and crude protein yields in China (*p*<0.001). Particularly, FM had the most substantial positive impact on lucerne crude protein yields, which is because it has the synergistic effects of mineral and manure fertilizers. Long-term utilization of mineral fertilizers may accelerate humus mineralization and degradation of soil quality (Menšík et al., 2018), while manure fertilizers could improve soil structure and fertility by adding organic matter (Minghao, 2023), the improvement of soil structure and quality may be the reason why lucerne with a higher CP content under FM fertilization.

Furthermore, all fertilizer types substantially reduced the ADF and NDF contents, indicating improved nutritional quality of lucerne and consistent with previous studies (Parsons et al., 2009). Interestingly, the meta-analysis revealed that unbalanced mineral fertilizer applications had a more pronounced effect on reducing ADF and NDF than balanced applications. An earlier study reported that combined nitrogen and phosphorus (NP) fertilizer application significantly reduced ADF, while combined phosphorus and potassium (PK) fertilizer application decreased NDF (Wan et al., 2022), suggesting possible interactions between different mineral fertilizers in reducing ADF and NDF in lucerne (**Table 4**), an effect potentially mitigated by balanced fertilizer application. In contrast, balanced mineral fertilizer applications increased the ash content and RFV more than unbalanced mineral fertilizer applications. In previous studies it was proposed that RFV did not improve with K fertilization, while unbalanced fertilization of N and P increased RFV (Yost et al., 2011; Zhang et al., 2020). Therefore, these results from different treatments may be contributed to the specific function of N and P fertilizer, which caused contrast changes in RFV.

**Table 4:** Meta-analysis on the effect of mineral fertilizer addition on ADF, NDF, and RFV.

	*RR*_++_	95% CI	95%+CI	Random effects model	
				*z*	*p*-value
ADF	–0.30	–0.37	–0.24	–8.71	<.001
NDF	–0.22	–0.29	–0.16	–6.37	<.001
RFV	0.37	0.31	0.43	11.55	<.001

ADF, acid detergent fiber; NDF, neutral detergent fiber; RFV, relative feed value.

Traditionally, an inverse relationship is observed between yield and quality, especially regarding fiber content in lucerne (Volenec et al., 1987; Feng et al., 2022). However, our findings present a more nuanced scenario, where strategic fertilization not only increases lucerne yield but also enhances certain quality indicators, which can be attributed to the application of mineral nutrients. Additionally, the incorporation of organic matter, particularly FM treatments, improves soil structure and microbial activity (Ananda et al., 2021; Hakl et al., 2021). Because manure fertilizer could increase the amount of soil organic carbon, which is essential to the growth of fungi, resulting in the change of soil microbial activity (Wen et al., 2020). In turn, soil microorganisms improve soil structure by producing specific exudates enhancing soil aggregate (Costa et al., 2018). This is in line with a previous study, which reported that manure increased soil organic carbon and soil N. Therefore, the difference in yield and nutritive value of lucerne could be attributed to soil changes in response to different fertilization (Zavattaro et al., 2017; Hakl et al., 2021). These changes facilitate an overall enhancement in lucerne digestibility and quality (Hakl et al., 2016; Feng et al., 2022; Yahaya et al., 2023).

### 4.3 Effect of environmental and agronomic factors on lucerne yield and nutritional quality traits under fertilization

The yield and nutritional quality of lucerne, including lucerne, are influenced by various environmental and agronomic factors, such as average annual temperature, precipitation, sowing date, and fertilization methods (Macolino et al., 2013; Gardarin et al., 2014; Lee et al., 2017).

Our meta-analysis indicated that MAT significantly affected lucerne yield and quality, while MAP primarily influenced quality traits (**Figure 6**). This is consistent with prior research asserting that temperature holds a more potent influence over yield than precipitation, affirming temperature as a critical determinant of lucerne yield (Ta et al., 2020; Wan et al., 2022). Specifically, higher temperatures were associated with decreased lucerne yield, CP, CPY, and RFV and increased ADF and NDF. This suggests that while temperature can promote lucerne growth and photosynthesis within an optimal range, exceeding the optimal temperature may negatively impact yield and quality. Liang et al. (2013) argued that increased temperature generally enhanced lucerne yield and CP content by promoting growth, photosynthesis, and accumulation of dry matter. Furthermore, with global warming, MAT will rise gradually, the yield and quality of lucerne will inevitably be affected, so it is necessary to determine the appropriate fertilization strategy according to the situation.

Soil properties also played a role in lucerne yield and quality. Positive correlations occurred between initial soil TN and lucerne yield and between initial soil TN and CPY, with a negative correlation between initial soil TN and NDF. These correlations imply that increased nitrogen might enhance nodule formation and thus improve yield and quality (Oliveira et al., 2004). Soil pH also affected lucerne yield and quality. Increased pH within an optimal range generally improved lucerne yield and quality traits, potentially promoting root growth and nodulation with N-fixing bacteria (Moreira and Fageria, 2010).

Planting system parameters such as sowing date and harvest frequency affected lucerne yield and quality (Wang et al., 2023). Previous studies indicated that delaying the sowing date of lucerne might lead to a reduction in overall yield and RFV, while simultaneously increasing ADF and NDF contents (Undersander, 2011). This suggests that sowing lucerne earlier in the season may be beneficial for both yield and nutritional quality. Increased harvest frequency and lucerne stand age improved quality traits by decreasing ADF and increasing CP, contrary to typical lucerne growth and development patterns. As lucerne stand ages, lucerne yield typically increases due to the deeper root system, enhanced water and nutrient absorption, and accelerated growth rate, while quality declines due to the reduced nutritional value (Nagasuga et al., 2011; Palmonari et al., 2014; Grev et al., 2020). However, this trend is contingent on adequate water supply and proper stand maintenance. Without these, lucerne yield often peaks at 3–5 years before declining (Fang et al., 2021; Wang et al., 2023; Zhao et al., 2023). These findings suggest that careful management of sowing date and harvest frequency can optimize lucerne yield and quality outcomes.

In general, the optimal harvest stage for lucerne is typically when it reaches the early to the mid-bloom stage, as this period offers a balance between maximizing yield and maintaining high nutritive quality regarding an optimal CP content while keeping lower fiber content (Parsons et al., 2009; Wang et al., 2023). Increasing the frequency of harvest generally leads to higher CP content and lower fiber content, enhancing the lucerne quality. However, this comes at the cost of reduced yield per harvest due to the shorter growth periods. In contrast, less frequent harvesting allows for greater biomass accumulation, leading to higher yields but potentially lower nutritive value due to increased fiber content. Thus, it is critical for farmers to carefully balance harvest frequency and timing based on their specific yield and quality objectives.

Correlations between various lucerne quality traits and yield revealed significant positive correlations between lucerne yield and CP, CPY, and NDF and negative correlations between RFV and both ADF and NDF. A previous study also reported decreased quality traits as lucerne yield increases (Undersander, 2011). Consequently, it is crucial to balance yield and quality during lucerne cultivation to achieve the best outcomes.

### 4.4 Research implications, limitations and future perspectives

Our study offers critical insights into the specific impacts of various fertilization types on lucerne yield and nutritional quality across China. We discovered that the combined application of manure and mineral fertilizer significantly enhances both the yield and crude protein yield of lucerne, outperforming other fertilization strategies. This indicates that strategic and balanced fertilizer use is vital for optimizing lucerne cultivation. Additionally, we have highlighted that environmental factors, particularly MAT, play a significant role in determining lucerne yield and quality. Interestingly, our findings revealed a negative correlation between MAT and key parameters such as lucerne yield, CP, CPY, and RFV. On the agronomic front, we observed a negative correlation between sowing date and yield and quality traits, underscoring the importance of early sowing for better yield and quality outcomes. Therefore, we suggested that the combined application of manure and mineral fertilizer could be better utilized to obtain high yield and good quality lucerne in China. In addition, MAT and sowing date are vital factors influencing yield and quality of lucerne under fertilization.

While our meta-analysis offers valuable insights into the effects of various fertilization regimes on lucerne yield and quality within China, these results are specific to the regional conditions analyzed. The applicability of our conclusions to other regions or under different environmental conditions remains to be verified. Additionally, our analysis focused on comparing different types of fertilizers rather than establishing optimal fertilization rates for each type. This limitation points to the need for more detailed quantitative studies that can determine the most effective fertilization gradients for maximizing both yield and quality of lucerne globally.

Future research should explore the differential impacts of fertilization types under varied climatic and soil conditions to determine more tailored fertilization strategies. Moreover, there is a significant need to examine the long-term effects of these fertilization practices on soil health and lucerne sustainability. This could include studying the cumulative effects of mineral and organic fertilizers on soil structure, microbial communities, and overall ecosystem services provided by lucerne fields.

## 5 Conclusions

This meta-analysis demonstrated the profound impact of various fertilization types on the yield and nutritional quality of lucerne. Particularly, the combination of mineral and manure fertilizer application significantly enhances lucerne yield and crude protein yield. It optimizes the incorporation of critical constituents like ash and relative feed value, while also elevating nutritional quality by reducing acid detergent fiber and neutral detergent fiber content. Furthermore, this research indicated a substantial influence of climatic variables, primarily the mean annual temperature, on lucerne yield and quality. It is noteworthy that the interplay between soil characteristics such as N content and pH level, in conjunction with agronomic factors like sowing date and harvest frequency, also significantly influences lucerne yield and quality.

Hence, the combination of mineral and manure fertilizer can be regarded as the primary fertilization institution of lucerne in China. Furthermore, it is of significance to choose the optimal sowing date and artificially regulate the system temperature. We recognized this meta-analysis involved some limitations, therefore, these conclusions should be examined in various regions or different environmental conditions in China to prove the reality of these conclusions. However, it is important to note that our results are rooted in the context of China, implying that regional disparities may occur. This highlights the need for future research to factor in location-specific parameters and delve deeper into the multifaceted interactions between diverse environmental and agronomic variables.

## Data availability statement

The raw data supporting the conclusions of this article will be made available by the authors, without undue reservation.

## Author contributions

JZ: Conceptualization, Data curation, Formal analysis, Validation, Visualization, Writing – original draft, Writing – review & editing. YM: Conceptualization, Validation, Visualization, Writing – review & editing. GW: Conceptualization, Writing – review & editing. DL: Conceptualization, Data curation, Writing – review & editing. QC: Conceptualization, Validation, Writing – review & editing. KS: Conceptualization, Writing – review & editing. MM: Writing – review & editing. MS: Writing – review & editing. FA: Writing – review & editing. ER: Writing – review & editing. YX: Writing – review & editing. QZ: Data curation, Formal analysis, Validation, Writing – review & editing. YL: Conceptualization, Data curation, Formal analysis, Funding acquisition, Writing – review & editing. YS: Funding acquisition, Resources, Writing – review & editing.
