# Supplementary material for: Enhancing lucerne (Medicago sativa) yield and nutritional quality: a meta-analysis of fertilization types and environmental factors in China
Source: Front Plant Sci. 2024 Jul 9;15:1405180. doi: 10.3389/fpls.2024.1405180 (PMC11263078; doi:10.3389/fpls.2024.1405180)
Supplement: Supplementary file 1 [file DataSheet_1.docx]

**Table S1.** Summary of specific information on fertilizer application rates (kg hm^-2^) for the meta-analysis.

|  | UCF | CF | BioF | FM | M |
| --- | --- | --- | --- | --- | --- |
| Min | 15.00 | 15.00 | 180.00 | 60.00 | 37.50 |
| Max | 900.00 | 562.50 | 600.00 | 180.00 | 360.00 |
| Median | 120.00 | 107.5 | 375.00 | 120.00 | 180.00 |
| Mean | 174.88 | 142.21 | 388.00 | 109.09 | 195.36 |
| SD | 149.07 | 106.45 | 162.35 | 52.43 | 122.99 |
| CV | 0.85 | 0.75 | 0.42 | 0.48 | 0.63 |

Note: UCF, Unbalanced mineral fertilization of N, P, or K; CF, Balanced mineral fertilization of N, P, and K; BioF, Biological fertilizer; FM, Manure and mineral fertilizers; M, Manure only. Fertilizers include nitrogen (N), phosphorus (P), and potassium (K); UCF is the unbalanced application of one or two types of mineral fertilizer (N only, P only, K only, N and P, N and K, and P and K); CF is the balanced mineral fertilization of N, P, and K.

**Fig. S1** Linear regression between yield and relative feeding value (RFV) under (a) treatment and (b) control group.


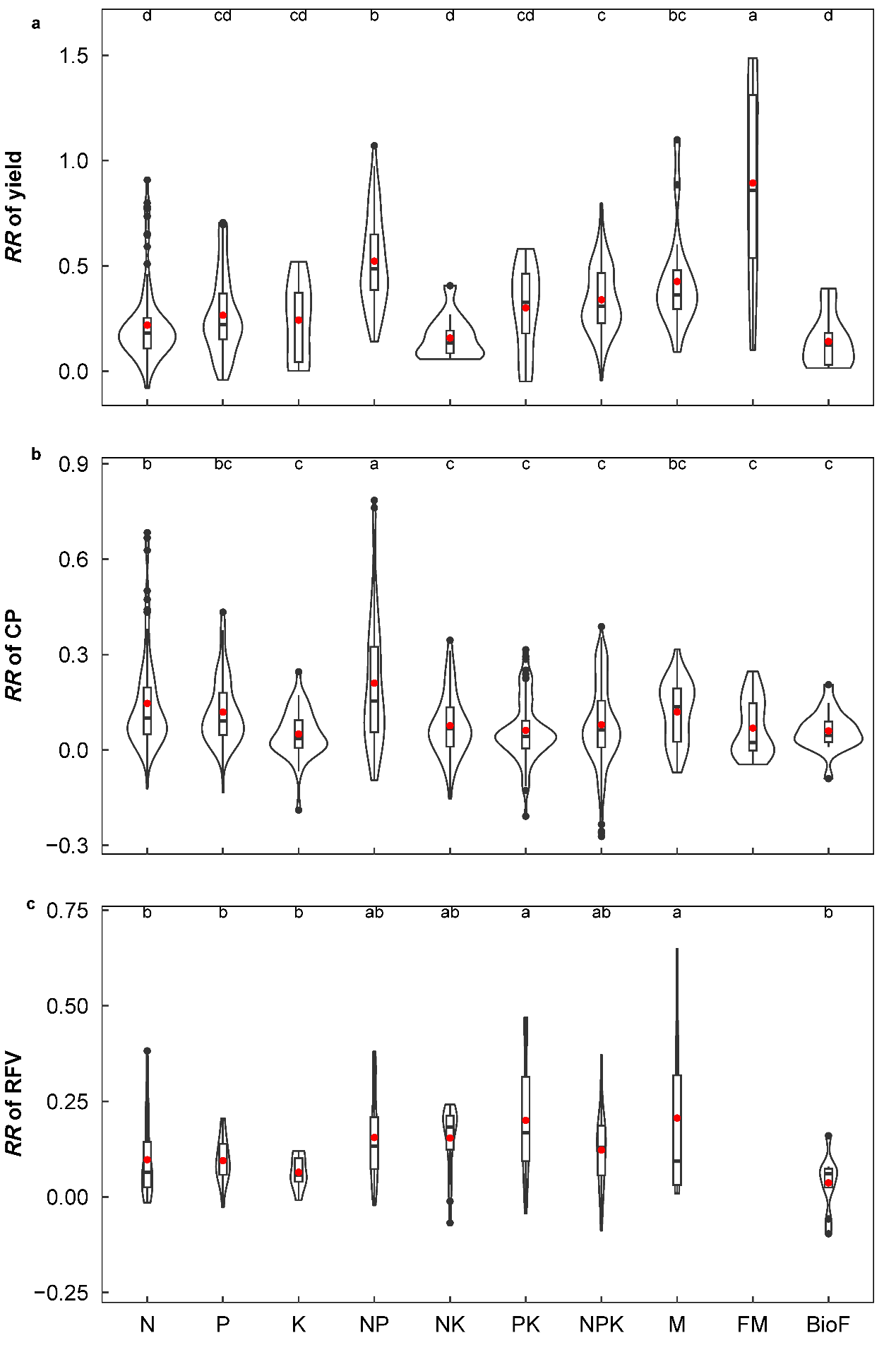


**Fig. S2** Overview of response ratio of lucerne (a) yield, (b) crude protein (CP), and (c) relative feed value (RFV) among different fertilizer types, which include N, P, K, NP, NK, PK, NPK, M, FM, BioF. Horizontal line and red dot indicate the median and average values, box limits represent the 25th and 75th percentiles (lower and upper limits, respectively), and vertical bars represent the 5th and 95th percentiles. Note the different scales between graphs. Statistical significance between groups are indicated by letters at 0.05 level.
